# Supplementary material for: Obesity Risk-Factor Variation Based on Island Clusters: A Secondary Analysis of Indonesian Basic Health Research 2018
Source: Nutrients. 2022 Feb 24;14(5):971. doi: 10.3390/nu14050971 (PMC8912714; doi:10.3390/nu14050971)
Supplement: Supplementary file 1 [file nutrients-14-00971-s001.zip › nutrients-1580409-supplementary.pdf]

Supplementary Material

Supplementary Tables

Table S1A. Obesity prevalence by provinces in Indonesia from RISKESDAS 2018

Island cluster	Province	Obesity Status	
		Non-obese	Obese
Sumatera	Aceh	61.70	38.30
	North Sumatera	59.40	40.60
	West Sumatera	66.50	33.50
	Riau	61.90	38.10
	Jambi	69.10	30.90
	South Sumatera	69.60	30.40
	Bengkulu	67.00	33.00
	Lampung	70.40	29.60
	Kepulauan Bangka Belitung	62.60	37.40
	Kepulauan Riau	60.20	39.80
Java	DKI Jakarta	54.40	45.60
	West Java	63.30	36.70
	Central Java	66.70	33.30
	DI Yogyakarta	65.40	34.60
	East Java	65.40	36.10
	Banten	64.70	35.30
Bali and Nusa Tenggara	Bali	61.20	38.80
	Nusa Tenggara Barat	73.30	26.70
	Nusa Tenggara Timur	80.90	19.10
Kalimantan	West Kalimantan	69.70	30.30
	Central Kalimantan	68.10	31.90
	South Kalimantan	67.00	33.00
	East Kalimantan	55.90	44.10
	North Kalimantan	59.80	40.20
Sulawesi	North Sulawesi	53.40	46.60
	Central Sulawesi	65.50	34.50
	South Sulawesi	67.90	32.10
	Southeast Sulawesi	67.60	32.40
	Gorontalo	60.80	39.20
	West Sulawesi	68.60	31.40
Maluku	Maluku	67.00	33.00

	North Maluku	62.10	37.90
Papua	West Papua	60.40	39.60
	Papua	65.00	35.00

Table S2A. Odd ratio of obesity based on the risk factors in Indonesia from RISKESDAS 2018

**Variables**		**Sumatera**	**Java**	**Bali and Nusa Tenggara**	**Kalimantan**	**Sulawesi**	**Maluku**	**Papua**
Constant	Odd Ratio	0.2	0.176	0.083	0.11	0.13	0.104	0.282
**Location (X1) -> Rural**								
Urban	Odd Ratio	**1.271**	1.372	**1.627**	1.442	1.348	1.497	1.287
	(95% CI)	(1.269-1.273)***	(1.37-1.374)***	(1.62-1.634)***	(1.437-1.447)***	(1.343-1.352)***	(1.483-1.51)***	(1.278-1.297)***
**Sex (X2) -> Male**								
Female	Odd Ratio	1.804	1.94	1.79	1.901	1.63	2.169	1.703
	(95% CI)	(1.8-1.808)***	(1.937-1.943)***	(1.779-1.801)***	(1.892-1.91)***	(1.623-1.638)***	(2.142-2.196)***	(1.689-1.718)***
**Marital Status (X3) -> Not married**								
Married	Odd Ratio	2.724	2.321	2.503	2.193	2.66	3.312	2.457
	(95% CI)	(2.717-2.731)***	(2.317-2.326)***	(2.487-2.52)***	(2.181-2.205)***	(2.646-2.673)***	(3.267-3.358)***	(2.429-2.485)***
Divorce	Odd Ratio	1.884	1.683	2.032	1.251	1.742	2.218	2.28
	(95% CI)	(1.873-1.895)***	(1.676-1.689)***	(2.004-2.06)***	(1.235-1.267)***	(1.723-1.762)***	(2.147-2.291)***	(2.213-2.349)***
Widow	Odd Ratio	2.024	1.85	1.837	1.358	1.841	2.797	2.143
	(95% CI)	(2.016-2.033)***	(1.845-1.856)***	(1.817-1.858)***	(1.345-1.372)***	(1.826-1.856)***	(2.734-2.861)***	(2.101-2.185)***
**Age (X4) -> <= 47 years**								
48-63 years	Odd Ratio	0.788	0.724	0.806	0.745	0.762	0.809	0.97
	(95% CI)	(0.787-0.79)***	(0.724-0.725)***	(0.802-0.81)***	(0.742-0.748)***	(0.759-0.765)***	(0.801-0.818)***	(0.962-0.979)***
>=64 years	Odd Ratio	0.336	0.273	0.331	0.352	0.335	0.359	0.349
	(95% CI)	(0.335-0.338)***	(0.273-0.274)***	(0.328-0.335)***	(0.349-0.355)***	(0.333-0.338)***	(0.352-0.366)***	(0.341-0.357)***
**Education level (X5) -> High**								
Low	Odd Ratio	0.753	0.754	0.868	0.807	0.877	0.825	0.63
	(95% CI)	(0.75-0.756)***	(0.752-0.756)***	(0.861-0.874)***	(0.801-0.813)***	(0.872-0.883)***	(0.812-0.839)***	(0.621-0.639)***
**Occupation (X6) -> Unemployed**								
Student	Odd Ratio	0.777	0.814	0.866	0.754	0.671	0.646	0.788
	(95% CI)	(0.773-0.781)***	(0.811-0.816)***	(0.857-0.876)***	(0.747-0.761)***	(0.665-0.677)***	(0.631-0.661)***	(0.774-0.803)***
Government officer/ military/ police	Odd Ratio	1.475	1.429	1.587	1.479	1.28	1.826	1.565

	(95% CI)	(1.468-1.482)***	(1.424-1.434)***	(1.572-1.603)***	(1.467-1.491)***	(1.271-1.29)***	(1.796-1.856)***	(1.543-1.587)***
Private company officer	Odd Ratio	1.119	1.078	1.541	1.251	1.126	1.609	0.95
	(95% CI)	(1.115-1.124)***	(1.076-1.08)***	(1.53-1.552)***	(1.244-1.258)***	(1.118-1.133)***	(1.577-1.642)***	(0.936-0.964)***
Entrepreneur	Odd Ratio	1.295	1.302	1.775	1.251	1.235	1.737	1.084
	(95% CI)	(1.291-1.298)***	(1.3-1.304)***	(1.764-1.786)***	(1.244-1.257)***	(1.229-1.241)***	(1.711-1.762)***	(1.072-1.097)***
Farmer	Odd Ratio	0.7	0.627	0.648	0.641	0.639	0.754	0.611
	(95% CI)	(0.698-0.701)***	(0.626-0.628)***	(0.644-0.652)***	(0.637-0.644)***	(0.636-0.643)***	(0.744-0.763)***	(0.605-0.617)***
Fisherman	Odd Ratio	0.68	1.008	1.337	0.964	0.691	0.571	0.505
	(95% CI)	(0.673-0.688)***	(0.999-1.017)	(1.311-1.364)***	(0.948-0.98)***	(0.682-0.699)***	(0.55-0.592)***	(0.49-0.52)***
Labor/ driver/ housekeeper	Odd Ratio	0.874	0.831	0.983	0.857	0.924	1.281	0.763
	(95% CI)	(0.871-0.878)***	(0.83-0.833)***	(0.975-0.991)***	(0.85-0.864)***	(0.917-0.93)***	(1.255-1.308)***	(0.747-0.779)***
Others	Odd Ratio	1.138	1.07	1.146	1.07	1.037	1.394	1.312
	(95% CI)	(1.134-1.142)***	(1.068-1.073)***	(1.136-1.155)***	(1.063-1.077)***	(1.031-1.042)***	(1.376-1.413)***	(1.293-1.333)***
**Mental emotional status (X7) -> Without mental emotional**								
With mental emotional	Odd Ratio	1.027	0.982	0.934	0.973	0.906	0.779	1.116
	(95% CI)	(1.024-1.03)***	(0.98-0.984)***	(0.929-0.94)***	(0.967-0.978)***	(0.902-0.91)***	(0.769-0.789)***	(1.104-1.128)***
**Sweet food (X8) -> <3 times / month**								
>1 time/day	Odd Ratio	0.987	0.906	1.048	0.888	1.028	1.235	1.012
	(95% CI)	(0.983-0.99)***	(0.904-0.908)***	(1.039-1.057)***	(0.882-0.895)***	(1.021-1.035)***	(1.213-1.256)***	(0.999-1.025)
**Sugar sweetened beverages (X9) -> <3 times / month**								
>1 time/day	Odd Ratio	0.807	0.809	0.824	0.809	0.832	0.744	0.858
	(95% CI)	(0.804-0.81)***	(0.807-0.81)***	(0.819-0.83)***	(0.803-0.815)***	(0.827-0.837)***	(0.731-0.756)***	(0.848-0.869)***
**Food high in salt (X10) -> <3 times / month**								
>1 time/day	Odd Ratio	0.858	0.916	1.173	1.002	0.885	1.264	0.86
	(95% CI)	(0.855-0.861)***	(0.915-0.918)***	(1.161-1.185)***	(0.996-1.009)	(0.879-0.891)***	(1.24-1.287)***	(0.846-0.874)***
**High-fat food (X11) -> <3 times / month**								
>1 time/day	Odd Ratio	1.183	1.158	1.406	1.245	1.482	1.133	1.141
	(95% CI)	(1.179-1.187)***	(1.156-1.161)***	(1.397-1.416)***	(1.238-1.253)***	(1.473-1.491)***	(1.115-1.152)***	(1.126-1.156)***

**Meat food (X12) -> <3 times / month**								
>1 time/day	Odd Ratio	1.356	0.887	1.088	1.024	1.01	0.982	1.104
	(95% CI)	(1.343-1.369)***	(0.883-0.891)***	(1.067-1.109)***	(1.009-1.04)***	(0.997-1.023)	(0.964-1.001)	(1.078-1.13)***
**Carbonated drinks (X13) -> <3 times / month**								
>1 time/day	Odd Ratio	0.984	0.902	0.839	0.868	1.525	1.609	1.809
	(95% CI)	(0.971-0.997)*	(0.896-0.908)***	(0.816-0.863)***	(0.849-0.887)***	(1.499-1.551)***	(1.552-1.668)***	(1.758-1.861)***
**Energy drink (X14) -> <3 times / month**								
>1 time/day	Odd Ratio	0.953	1.171	0.561	0.849	0.88	0.928	1.01
	(95% CI)	(0.939-0.967)***	(1.162-1.18)***	(0.54-0.583)***	(0.832-0.867)***	(0.862-0.897)***	(0.898-0.959)***	(0.985-1.036)
**Instant food (X15) -> <3 times / month**								
>1 time/day	Odd Ratio	0.87	1.038	0.968	0.827	0.78	0.926	0.992
	(95% CI)	(0.864-0.876)***	(1.034-1.042)***	(0.953-0.984)***	(0.818-0.837)***	(0.773-0.787)***	(0.907-0.946)***	(0.975-1.009)
**FV consumption (X16) -> Adequate**								
Inadequate	Odd Ratio	0.894	0.83	0.799	0.77	0.96	0.775	0.724
	(95% CI)	(0.89-0.898)***	(0.828-0.832)***	(0.793-0.805)***	(0.765-0.776)***	(0.953-0.966)***	(0.764-0.786)***	(0.715-0.732)***
**Smoking behavior (X17) -> Never smoke**								
Quitted	Odd Ratio	1.143	1.065	1.077	1.12	0.917	1.501	1.063
	(95% CI)	(1.138-1.148)***	(1.063-1.068)***	(1.067-1.087)***	(1.112-1.129)***	(0.91-0.923)***	(1.469-1.533)***	(1.043-1.083)***
Currently smoke	Odd Ratio	0.794	0.693	0.685	0.709	0.664	0.822	0.779
	(95% CI)	(0.792-0.797)***	(0.692-0.694)***	(0.68-0.69)***	(0.706-0.713)***	(0.661-0.668)***	(0.811-0.833)***	(0.771-0.786)***
**Physical activity (X18) -> Active**								
Inactive	Odd Ratio	1.009	1.099	1.093	1.113	1.028	1.026	1.05
	(95% CI)	(1.008-1.011)***	(1.098-1.1)***	(1.089-1.098)***	(1.109-1.117)***	(1.024-1.031)***	(1.017-1.035)***	(1.043-1.057)***
**Alcohol consumption (X19) -> No**								
Yes	Odd Ratio	0.835	0.75	0.763	1.085	0.954	1.132	1.02
	(95% CI)	(0.83-0.839)***	(0.747-0.753)***	(0.758-0.768)***	(1.075-1.095)***	(0.948-0.96)***	(1.115-1.151)***	(1.005-1.036)*
**Blood pressure (X20) -> Normal**								
Prehypertension	Odd Ratio	2.259	2.438	2.745	2.434	2.512	2.413	2.059
	(95% CI)	(2.254-2.265)***	(2.435-2.442)***	(2.731-2.759)***	(2.421-2.446)***	(2.502-2.522)***	(2.388-2.439)***	(2.042-2.076)***

Hypertension stage 1	Odd Ratio	3.94	4.382	4.765	4.619	4.049	4.695	3.654
	(95% CI)	(3.929-3.95)***	(4.375-4.389)***	(4.736-4.793)***	(4.594-4.644)***	(4.031-4.068)***	(4.638-4.752)***	(3.618-3.69)***
Hypertension stage 2	Odd Ratio	5.984	6.556	7.398	6.731	5.684	6.358	5.165
	(95% CI)	(5.966-6.003)***	(6.544-6.568)***	(7.347-7.448)***	(6.692-6.771)***	(5.655-5.714)***	(6.266-6.451)***	(5.099-5.232)***

†CI: Confidence of interval

***: Very significant

Table S3A. Effect size of odd ratios for marital status variable

Island cluster comparison	Cohen's d				Cliff's Delta			
	D Estimation	95 CI		Effect Size	D Estimation	95 CI		Effect Size
		Lower	Upper			Lower	Upper	
Sumatera - Java	0.5363	-1.7710	2.8437	medium	0.5556	-0.6148	0.9618	large
Sumatera - Bali_Nusa Tenggara	0.3756	-1.9113	2.6624	small	0.5556	-0.6148	0.9618	large
Sumatera - Kalimantan	0.7767	-1.5742	3.1275	medium	0.5556	-0.6148	0.9618	large
Sumatera - Sulawesi	0.5010	-1.8012	2.8033	medium	0.5556	-0.6148	0.9618	large
Sumatera - Maluku	0.0138	-2.2532	2.2808	negligible	0.1111	-0.8227	0.8828	negligible
Sumatera - Papua	-0.1473	-2.4173	2.1227	negligible	-0.1111	-0.8828	0.8227	negligible
Java - Bali_Nusa Tenggara	-0.0861	-2.3541	2.1819	negligible	0.1111	-0.5660	0.6987	negligible
Java - Kalimantan	0.3679	-1.9182	2.6539	small	0.3333	-0.5107	0.8502	medium
Java - Sulawesi	0.0613	-2.2062	2.3288	negligible	0.3333	-0.5107	0.8502	medium
Java - Maluku	-0.4515	-2.7472	1.8442	small	-0.1111	-0.7930	0.6943	negligible
Java - Papua	-0.9087	-3.2897	1.4724	large	-0.3333	-0.9495	0.8121	medium
Bali_Nusa Tenggara - Kalimantan	0.3955	-1.8935	2.6846	small	0.5556	-0.6148	0.9618	large
Bali_Nusa Tenggara - Sulawesi	0.1268	-2.1424	2.3960	negligible	0.5556	-0.6148	0.9618	large
Bali_Nusa Tenggara - Maluku	-0.3253	-2.6072	1.9566	small	-0.1111	-0.7930	0.6943	negligible
Bali_Nusa Tenggara - Papua	-0.5929	-2.9091	1.7234	medium	-0.3333	-0.9495	0.8121	medium
Kalimantan - Sulawesi	-0.2660	-2.5430	2.0109	small	-0.5556	-0.9618	0.6148	large
Kalimantan - Maluku	-0.6934	-3.0275	1.6407	medium	-0.5556	-0.9618	0.6148	large

Kalimantan - Papua	-1.0542	-3.4735	1.3651	large	-0.3333	-0.9495	0.8121	medium
Sulawesi - Maluku	-0.4406	-2.7349	1.8537	small	-0.1111	-0.7930	0.6943	negligible
Sulawesi - Papua	-0.7346	-3.0768	1.6075	medium	-0.3333	-0.9495	0.8121	medium
Maluku - Papua	-0.1392	-2.4089	2.1305	negligible	-0.1111	-0.8828	0.8227	negligible

Table S3B. Effect size of odd ratios for age variable

Island cluster comparison	Cohen's d				Cliff's Delta			
	D Estimation	95 CI		Effect Size	D Estimation	95 CI		Effect Size
		Lower	Upper			Lower	Upper	
Sumatera - Java	0.2226	-4.0934	4.5385	small	0.5000	-0.8416	0.9811	large
Sumatera - Bali_Nusa Tenggara	0.1625	-4.1472	4.4723	negligible	0.5000	-0.8416	0.9811	large
Sumatera - Kalimantan	0.1913	-4.1212	4.5037	negligible	0.0000	-0.9740	0.9740	negligible
Sumatera - Sulawesi	0.1432	-4.1649	4.4514	negligible	0.5000	-0.8416	0.9811	large
Sumatera - Maluku	0.0753	-4.2289	4.3795	negligible	0.5000	-0.8416	0.9811	large
Sumatera - Papua	-0.0594	-4.3630	4.2442	negligible	0.0000	-0.9740	0.9740	negligible
Java - Bali_Nusa Tenggara	-0.0628	-4.3666	4.2409	negligible	-0.5000	-0.9811	0.8416	large
Java - Kalimantan	-0.0607	-4.3644	4.2429	negligible	0.0000	-0.9740	0.9740	negligible
Java - Sulawesi	-0.0927	-4.3977	4.2123	negligible	-0.5000	-0.9811	0.8416	large
Java - Maluku	-0.1495	-4.4582	4.1591	negligible	-0.5000	-0.9811	0.8416	large
Java - Papua	-0.2686	-4.5906	4.0534	small	-0.5000	-0.9811	0.8416	large
Bali_Nusa Tenggara - Kalimantan	0.0097	-4.2929	4.3124	negligible	0.0000	-0.9740	0.9740	negligible
Bali_Nusa Tenggara - Sulawesi	-0.0273	-4.3301	4.2756	negligible	0.0000	-0.9740	0.9740	negligible
Bali_Nusa Tenggara - Maluku	-0.0879	-4.3927	4.2168	negligible	-0.5000	-0.9811	0.8416	large
Bali_Nusa Tenggara - Papua	-0.2126	-4.5274	4.1022	small	-0.5000	-0.9811	0.8416	large
Kalimantan - Sulawesi	-0.0414	-4.3445	4.2618	negligible	0.0000	-0.9740	0.9740	negligible
Kalimantan - Maluku	-0.1083	-4.4141	4.1975	negligible	0.0000	-0.9740	0.9740	negligible

Kalimantan - Papua	-0.2430	-4.5615	4.0755	small	0.0000	-0.9740	0.9740	negligible
Sulawesi - Maluku	-0.0649	-4.3687	4.2388	negligible	0.0000	-0.9740	0.9740	negligible
Sulawesi - Papua	-0.1962	-4.5092	4.1168	negligible	-0.5000	-0.9811	0.8416	large
Maluku - Papua	-0.1307	-4.4379	4.1766	negligible	-0.5000	-0.9811	0.8416	large

Table S3C. Effect size of odd ratios for level of education variable

Island cluster comparison	Cohen's d				Cliff's Delta			
	D Estimation	95 CI		Effect Size	D Estimation	95 CI		Effect Size
		Lower	Upper			Lower	Upper	
Sumatera - Java	-0.2572	-4.5776	4.0632	small	-0.5000	-0.9811	0.8416	large
Sumatera - Bali_Nusa Tenggara	-0.7920	-5.2602	3.6761	medium	-0.5000	-0.9811	0.8416	large
Sumatera - Kalimantan	-0.7248	-5.1664	3.7169	medium	-0.5000	-0.9811	0.8416	large
Sumatera - Sulawesi	-1.2149	-5.8977	3.4679	large	-0.5000	-0.9811	0.8416	large
Sumatera - Maluku	-1.6729	-6.6718	3.3260	large	-1.0000	-1.0000	0.8283	large
Sumatera - Papua	1.1568	-3.4918	5.8054	large	0.5000	-0.8416	0.9811	large
Java - Bali_Nusa Tenggara	-0.5163	-4.8901	3.8574	medium	-0.5000	-0.9811	0.8416	large
Java - Kalimantan	-0.4465	-4.8025	3.9094	small	-0.5000	-0.9811	0.8416	large
Java - Sulawesi	-0.8290	-5.3126	3.6547	large	-0.5000	-0.9811	0.8416	large
Java - Maluku	-1.2347	-5.9295	3.4600	large	-0.5000	-0.9811	0.8416	large
Java - Papua	1.3304	-3.4245	6.0852	large	0.5000	-0.8416	0.9811	large
Bali_Nusa Tenggara - Kalimantan	0.0771	-4.2271	4.3814	negligible	0.5000	-0.8416	0.9811	large
Bali_Nusa Tenggara - Sulawesi	-0.1837	-4.4955	4.1280	negligible	0.0000	-0.9740	0.9740	negligible
Bali_Nusa Tenggara - Maluku	-0.5524	-4.9364	3.8315	medium	-0.5000	-0.9811	0.8416	large
Bali_Nusa Tenggara - Papua	1.7839	-3.3030	6.8709	large	1.0000	-0.8283	1.0000	large
Kalimantan - Sulawesi	-0.2830	-4.6072	4.0411	small	0.0000	-0.9740	0.9740	negligible
Kalimantan - Maluku	-0.6648	-5.0848	3.7551	medium	-0.5000	-0.9811	0.8416	large

Kalimantan - Papua	1.7391	-3.3118	6.7900	large	1.0000	-0.8283	1.0000	large
Sulawesi - Maluku	-0.5120	-4.8846	3.8606	medium	-0.5000	-0.9811	0.8416	large
Sulawesi - Papua	2.3335	-3.2445	7.9114	large	1.0000	-0.8283	1.0000	large
Maluku - Papua	2.7479	-3.2510	8.7468	large	1.0000	-0.8283	1.0000	large

Table S3D. Effect size of odd ratios for occupational status variable

Island cluster comparison	Cohen's d				Cliff's Delta			
	D Estimation	95 CI		Effect Size	D Estimation	95 CI		Effect Size
		Lower	Upper			Lower	Upper	
Sumatera - Java	-0.0849	-1.1577	0.9880	negligible	-0.0313	-0.5764	0.5331	negligible
Sumatera - Bali_Nusa Tenggara	-0.5219	-1.6124	0.5686	medium	-0.2500	-0.7117	0.3625	small
Sumatera - Kalimantan	-0.0357	-1.1082	1.0367	negligible	-0.0625	-0.5963	0.5096	negligible
Sumatera - Sulawesi	0.0722	-1.0005	1.1450	negligible	0.1250	-0.4522	0.6284	negligible
Sumatera - Maluku	-0.4816	-1.5694	0.6063	small	-0.1250	-0.6622	0.4971	negligible
Sumatera - Papua	0.0008	-1.0715	1.0732	negligible	0.0625	-0.5041	0.5915	negligible
Java - Bali_Nusa Tenggara	-0.4422	-1.5276	0.6432	small	-0.2188	-0.6977	0.3953	small
Java - Kalimantan	0.0542	-1.0184	1.1268	negligible	0.0000	-0.5450	0.5450	negligible
Java - Sulawesi	0.1534	-0.9205	1.2274	negligible	0.1250	-0.4583	0.6330	negligible
Java - Maluku	-0.4146	-1.4984	0.6693	small	-0.1250	-0.6504	0.4813	negligible
Java - Papua	0.0810	-0.9918	1.1539	negligible	-0.0313	-0.5663	0.5224	negligible
Bali_Nusa Tenggara - Kalimantan	0.5075	-0.5820	1.5970	medium	0.2188	-0.3953	0.6977	small
Bali_Nusa Tenggara - Sulawesi	0.5756	-0.5188	1.6700	medium	0.2031	-0.4036	0.6858	small
Bali_Nusa Tenggara - Maluku	-0.0252	-1.0976	1.0472	negligible	0.0313	-0.5278	0.5714	negligible
Bali_Nusa Tenggara - Papua	0.5045	-0.5848	1.5938	medium	0.2188	-0.3886	0.6936	small
Kalimantan - Sulawesi	0.1110	-0.9623	1.1842	negligible	0.0625	-0.5041	0.5915	negligible
Kalimantan - Maluku	-0.4671	-1.5540	0.6198	small	-0.1563	-0.6749	0.4657	small

Kalimantan - Papua	0.0343	-1.0382	1.1068	negligible	0.0000	-0.5450	0.5450	negligible
Sulawesi - Maluku	-0.5281	-1.6191	0.5628	medium	-0.1875	-0.6867	0.4319	small
Sulawesi - Papua	-0.0670	-1.1397	1.0056	negligible	0.0000	-0.5450	0.5450	negligible
Maluku - Papua	0.4695	-0.6176	1.5565	small	0.1563	-0.4486	0.6629	small

Table S3E. Effect size of odd ratios for sweet food consumption variable

Island cluster comparison	Cohen's d				Cliff's Delta			
	D Estimation	95 CI		Effect Size	D Estimation	95 CI		Effect Size
		Lower	Upper			Lower	Upper	
Sumatera - Java	3.9928	-3.4507	11.4362	large	1.0000	-0.8283	1.0000	large
Sumatera - Bali_Nusa Tenggara	-3.6814	-10.7437	3.3808	large	-1.0000	-1.0000	0.8283	large
Sumatera - Kalimantan	5.1240	-3.7794	14.0274	large	1.0000	-0.8283	1.0000	large
Sumatera - Sulawesi	-3.0184	-9.3110	3.2741	large	-1.0000	-1.0000	0.8283	large
Sumatera - Maluku	-5.6110	-15.1697	3.9477	large	-1.0000	-1.0000	0.8283	large
Sumatera - Papua	0.7955	-3.6741	5.2650	medium	0.5000	-0.8416	0.9811	large
Java - Bali_Nusa Tenggara	-15.7735	-40.1512	8.6042	large	-1.0000	-1.0000	0.8283	large
Java - Kalimantan	1.8701	-3.2880	7.0282	large	1.0000	-0.8283	1.0000	large
Java - Sulawesi	-7.4808	-19.6471	4.6854	large	-1.0000	-1.0000	0.8283	large
Java - Maluku	-13.3527	-34.1157	7.4103	large	-1.0000	-1.0000	0.8283	large
Java - Papua	-0.2729	-4.5955	4.0498	small	0.0000	-0.9740	0.9740	negligible
Bali_Nusa Tenggara - Kalimantan	16.2105	-8.8218	41.2428	large	1.0000	-0.8283	1.0000	large
Bali_Nusa Tenggara - Sulawesi	-0.6887	-5.1171	3.7397	medium	0.0000	-0.9740	0.9740	negligible
Bali_Nusa Tenggara - Maluku	-4.5468	-12.6924	3.5989	large	-1.0000	-1.0000	0.8283	large
Bali_Nusa Tenggara - Papua	1.7184	-3.3161	6.7530	large	1.0000	-0.8283	1.0000	large
Kalimantan - Sulawesi	-8.4577	-22.0241	5.1087	large	-1.0000	-1.0000	0.8283	large
Kalimantan - Maluku	-14.2597	-36.3745	7.8550	large	-1.0000	-1.0000	0.8283	large

Kalimantan - Papua	-0.6248	-5.0312	3.7816	medium	0.0000	-0.9740	0.9740	negligible
Sulawesi - Maluku	-1.8220	-6.9401	3.2961	large	-1.0000	-1.0000	0.8283	large
Sulawesi - Papua	1.8377	-3.2934	6.9688	large	1.0000	-0.8283	1.0000	large
Maluku - Papua	2.4306	-3.2425	8.1037	large	1.0000	-0.8283	1.0000	large

Table S3F. Effect size of odd ratios for sugar-sweetened beverages consumption variable

Island cluster comparison	Cohen's d				Cliff's Delta			
	D Estimation	95 CI		Effect Size	D Estimation	95 CI		Effect Size
		Lower	Upper			Lower	Upper	
Sumatera - Java	-0.5412	-4.9219	3.8395	medium	-0.5000	-0.9811	0.8416	large
Sumatera - Bali_Nusa Tenggara	-0.4268	-4.7782	3.9246	small	-0.5000	-0.9811	0.8416	large
Sumatera - Kalimantan	-0.6687	-5.0900	3.7525	medium	-0.5000	-0.9811	0.8416	large
Sumatera - Sulawesi	-1.6816	-6.6872	3.3241	large	-1.0000	-1.0000	0.8283	large
Sumatera - Maluku	-1.1464	-5.7891	3.4962	large	-0.5000	-0.9811	0.8416	large
Sumatera - Papua	-0.6885	-5.1168	3.7398	medium	-0.5000	-0.9811	0.8416	large
Java - Bali_Nusa Tenggara	0.0277	-4.2752	4.3305	negligible	0.0000	-0.9740	0.9740	negligible
Java - Kalimantan	-0.1638	-4.4736	4.1461	negligible	-0.5000	-0.9811	0.8416	large
Java - Sulawesi	-1.1468	-5.7897	3.4961	large	-0.5000	-0.9811	0.8416	large
Java - Maluku	-0.4833	-4.8483	3.8817	small	0.0000	-0.9740	0.9740	negligible
Java - Papua	-0.2739	-4.5967	4.0489	small	0.0000	-0.9740	0.9740	negligible
Bali_Nusa Tenggara - Kalimantan	-0.1652	-4.4752	4.1448	negligible	-0.5000	-0.9811	0.8416	large
Bali_Nusa Tenggara - Sulawesi	-0.9642	-5.5100	3.5816	large	-0.5000	-0.9811	0.8416	large
Bali_Nusa Tenggara - Maluku	-0.3983	-4.7434	3.9468	small	0.0000	-0.9740	0.9740	negligible
Bali_Nusa Tenggara - Papua	-0.2665	-4.5882	4.0552	small	-0.5000	-0.9811	0.8416	large
Kalimantan - Sulawesi	-0.8947	-5.4075	3.6181	large	-0.5000	-0.9811	0.8416	large
Kalimantan - Maluku	-0.2325	-4.5496	4.0847	small	0.0000	-0.9740	0.9740	negligible

Kalimantan - Papua	-0.1348	-4.4423	4.1727	negligible	0.0000	-0.9740	0.9740	negligible
Sulawesi - Maluku	1.0110	-3.5583	5.5802	large	0.5000	-0.8416	0.9811	large
Sulawesi - Papua	0.5731	-3.8170	4.9631	medium	0.5000	-0.8416	0.9811	large
Maluku - Papua	0.0146	-4.2881	4.3173	negligible	0.0000	-0.9740	0.9740	negligible

Table S3G. Effect size of odd ratios for high-salt food consumption variable

Island cluster comparison	Cohen's d				Cliff's Delta			
	D Estimation	95 CI		Effect Size	D Estimation	95 CI		Effect Size
		Lower	Upper			Lower	Upper	
Sumatera - Java	-0.0123	-4.3150	4.2904	negligible	0.000	-0.974	0.974	negligible
Sumatera - Bali_Nusa Tenggara	-2.3398	-7.9238	3.2443	large	-1.000	-1.000	0.828	large
Sumatera - Kalimantan	-0.4308	-4.7830	3.9215	small	-0.500	-0.981	0.842	large
Sumatera - Sulawesi	2.3659	-3.2436	7.9753	large	1.000	-0.828	1.000	large
Sumatera - Maluku	-1.8731	-7.0337	3.2875	large	-1.000	-1.000	0.828	large
Sumatera - Papua	-0.1641	-4.4739	4.1458	negligible	0.000	-0.974	0.974	negligible
Java - Bali_Nusa Tenggara	-2.6021	-8.4486	3.2444	large	-1.000	-1.000	0.828	large
Java - Kalimantan	-0.5624	-4.9492	3.8245	medium	-0.500	-0.981	0.842	large
Java - Sulawesi	5.1171	-3.7771	14.0114	large	1.000	-0.828	1.000	large
Java - Maluku	-1.9841	-7.2399	3.2716	large	-1.000	-1.000	0.828	large
Java - Papua	-0.1726	-4.4832	4.1381	negligible	0.000	-0.974	0.974	negligible
Bali_Nusa Tenggara - Kalimantan	2.0991	-3.2590	7.4573	large	1.000	-0.828	1.000	large
Bali_Nusa Tenggara - Sulawesi	4.0394	-3.4620	11.5409	large	1.000	-0.828	1.000	large
Bali_Nusa Tenggara - Maluku	-0.0805	-4.3849	4.2239	negligible	0.000	-0.974	0.974	negligible
Bali_Nusa Tenggara - Papua	1.6460	-3.3322	6.6241	large	1.000	-0.828	1.000	large
Kalimantan - Sulawesi	3.2402	-3.3026	9.7830	large	1.000	-0.828	1.000	large

Kalimantan - Maluku	-1.6758	-6.6769	3.3254	large	-1.000	-1.000	0.828	large
Kalimantan - Papua	0.0990	-4.2062	4.4043	negligible	0.000	-0.974	0.974	negligible
Sulawesi - Maluku	-2.9891	-9.2491	3.2710	large	-1.000	-1.000	0.828	large
Sulawesi - Papua	-1.3839	-6.1739	3.4062	large	-1.000	-1.000	0.828	large
Maluku - Papua	1.4576	-3.3828	6.2979	large	1.000	-0.828	1.000	large

Table S3H. Effect size of odd ratios for high-fat food consumption variable

Island cluster comparison	Cohen's d				Cliff's Delta			
	D Estimation	95 CI		Effect Size	D Estimation	95 CI		Effect Size
		Lower	Upper			Lower	Upper	
Sumatera - Java	-0.0990	-4.4043	4.2063	negligible	0.0000	-0.9740	0.9740	negligible
Sumatera - Bali_Nusa Tenggara	-1.7946	-6.8902	3.3010	large	-1.0000	-1.0000	0.8283	large
Sumatera - Kalimantan	-1.2369	-5.9330	3.4592	large	-0.5000	-0.9811	0.8416	large
Sumatera - Sulawesi	-2.0567	-7.3767	3.2632	large	-1.0000	-1.0000	0.8283	large
Sumatera - Maluku	1.2061	-3.4714	5.8836	large	1.0000	-0.8283	1.0000	large
Sumatera - Papua	0.5999	-3.7985	4.9982	medium	0.5000	-0.8416	0.9811	large
Java - Bali_Nusa Tenggara	-1.9740	-7.2209	3.2729	large	-1.0000	-1.0000	0.8283	large
Java - Kalimantan	-1.4658	-6.3120	3.3803	large	-1.0000	-1.0000	0.8283	large
Java - Sulawesi	-2.2121	-7.6744	3.2502	large	-1.0000	-1.0000	0.8283	large
Java - Maluku	2.4289	-3.2425	8.1002	large	1.0000	-0.8283	1.0000	large
Java - Papua	0.7961	-3.6738	5.2659	medium	0.5000	-0.8416	0.9811	large
Bali_Nusa Tenggara - Kalimantan	0.8432	-3.6466	5.3330	large	0.5000	-0.8416	0.9811	large
Bali_Nusa Tenggara - Sulawesi	-0.4086	-4.7560	3.9387	small	-0.5000	-0.9811	0.8416	large
Bali_Nusa Tenggara - Maluku	3.0088	-3.2731	9.2907	large	1.0000	-0.8283	1.0000	large
Bali_Nusa Tenggara - Papua	2.1701	-3.2531	7.5932	large	1.0000	-0.8283	1.0000	large
Kalimantan - Sulawesi	-1.2152	-5.8981	3.4678	large	-0.5000	-0.9811	0.8416	large
Kalimantan - Maluku	3.0638	-3.2793	9.4070	large	1.0000	-0.8283	1.0000	large

Kalimantan - Papua	1.7191	-3.3160	6.7541	large	1.0000	-0.8283	1.0000	large
Sulawesi - Maluku	3.0851	-3.2819	9.4521	large	1.0000	-0.8283	1.0000	large
Sulawesi - Papua	2.3922	-3.2430	8.0275	large	1.0000	-0.8283	1.0000	large
Maluku - Papua	-0.2220	-4.5379	4.0939	small	0.0000	-0.9740	0.9740	negligible

Table S3I. Effect size of odd ratios for meat food consumption variable

Island cluster comparison	Cohen's d				Cliff's Delta			
	D Estimation	95 CI		Effect Size	D Estimation	95 CI		Effect Size
		Lower	Upper			Lower	Upper	
Sumatera - Java	1.0076	-3.5599	5.5752	large	0.5000	-0.8416	0.9811	large
Sumatera - Bali_Nusa Tenggara	0.0965	-4.2086	4.4017	negligible	0.0000	-0.9740	0.9740	negligible
Sumatera - Kalimantan	1.0280	-3.5500	5.6060	large	0.5000	-0.8416	0.9811	large
Sumatera - Sulawesi	0.7879	-3.6785	5.2544	medium	0.5000	-0.8416	0.9811	large
Sumatera - Maluku	1.1971	-3.4751	5.8692	large	0.5000	-0.8416	0.9811	large
Sumatera - Papua	-0.1900	-4.5023	4.1224	negligible	0.0000	-0.9740	0.9740	negligible
Java - Bali_Nusa Tenggara	-1.2017	-5.8766	3.4732	large	-0.5000	-0.9811	0.8416	large
Java - Kalimantan	-0.0500	-4.3534	4.2533	negligible	0.0000	-0.9740	0.9740	negligible
Java - Sulawesi	-0.4170	-4.7662	3.9321	small	-0.5000	-0.9811	0.8416	large
Java - Maluku	0.4767	-3.8866	4.8401	small	0.5000	-0.8416	0.9811	large
Java - Papua	-2.1970	-7.6451	3.2512	large	-1.0000	-1.0000	0.8283	large
Bali_Nusa Tenggara - Kalimantan	1.2664	-3.4478	5.9807	large	0.7500	-0.5776	0.9891	large
Bali_Nusa Tenggara - Sulawesi	0.9043	-3.6129	5.4215	large	0.5000	-0.8416	0.9811	large
Bali_Nusa Tenggara - Maluku	1.3571	-3.4152	6.1295	large	0.5000	-0.8416	0.9811	large
Bali_Nusa Tenggara - Papua	-0.3922	-4.7361	3.9516	small	-0.5000	-0.9811	0.8416	large
Kalimantan - Sulawesi	-0.4485	-4.8049	3.9079	small	-0.5000	-0.9811	0.8416	large
Kalimantan - Maluku	0.5463	-3.8359	4.9284	medium	0.0000	-0.9740	0.9740	negligible

Kalimantan - Papua	-2.5148	-8.2722	3.2426	large	-1.0000	-1.0000	0.8283	large
Sulawesi - Maluku	0.7605	-3.6950	5.2159	medium	0.5000	-0.8416	0.9811	large
Sulawesi - Papua	-1.7700	-6.8456	3.3057	large	-1.0000	-1.0000	0.8283	large
Maluku - Papua	-2.0248	-7.3164	3.2667	large	-1.0000	-1.0000	0.8283	large

Table S3J. Effect size of odd ratios for carbonated drink consumption variable

Island cluster comparison	Cohen's d				Cliff's Delta			
	D Estimation	95 CI		Effect Size	D Estimation	95 CI		Effect Size
		Lower	Upper			Lower	Upper	
Sumatera - Java	-0.6730	-5.0958	3.7498	medium	0.0000	-0.9740	0.9740	negligible
Sumatera - Bali_Nusa Tenggara	0.2245	-4.0917	4.5406	small	0.0000	-0.9740	0.9740	negligible
Sumatera - Kalimantan	-0.2527	-4.5725	4.0671	small	-0.5000	-0.9811	0.8416	large
Sumatera - Sulawesi	-1.5862	-6.5193	3.3469	large	-1.0000	-1.0000	0.8283	large
Sumatera - Maluku	-1.2696	-5.9858	3.4467	large	-1.0000	-1.0000	0.8283	large
Sumatera - Papua	-0.9947	-5.5556	3.5663	large	-0.5000	-0.9811	0.8416	large
Java - Bali_Nusa Tenggara	0.4306	-3.9216	4.7828	small	0.0000	-0.9740	0.9740	negligible
Java - Kalimantan	0.3880	-3.9549	4.7309	small	0.0000	-0.9740	0.9740	negligible
Java - Sulawesi	-1.4402	-6.2685	3.3881	large	-1.0000	-1.0000	0.8283	large
Java - Maluku	-1.1388	-5.7771	3.4995	large	-1.0000	-1.0000	0.8283	large
Java - Papua	-0.8961	-5.4096	3.6173	large	0.0000	-0.9740	0.9740	negligible
Bali_Nusa Tenggara - Kalimantan	-0.3211	-4.6514	4.0092	small	0.0000	-0.9740	0.9740	negligible
Bali_Nusa Tenggara - Sulawesi	-1.0095	-5.5780	3.5590	large	-0.5000	-0.9811	0.8416	large
Bali_Nusa Tenggara - Maluku	-1.1455	-5.7876	3.4966	large	-0.5000	-0.9811	0.8416	large
Bali_Nusa Tenggara - Papua	-0.9922	-5.5520	3.5675	large	-0.5000	-0.9811	0.8416	large
Kalimantan - Sulawesi	-1.4588	-6.3000	3.3824	large	-1.0000	-1.0000	0.8283	large
Kalimantan - Maluku	-1.1967	-5.8686	3.4752	large	-1.0000	-1.0000	0.8283	large

Kalimantan - Papua	-0.9430	-5.4785	3.5925	large	-0.5000	-0.9811	0.8416	large
Sulawesi - Maluku	-0.5098	-4.8818	3.8622	medium	0.0000	-0.9740	0.9740	negligible
Sulawesi - Papua	-0.4894	-4.8560	3.8772	small	0.0000	-0.9740	0.9740	negligible
Maluku - Papua	-0.1192	-4.4256	4.1873	negligible	0.0000	-0.9740	0.9740	negligible

Table S3K. Effect size of odd ratios for energy drink consumption variable

Island cluster comparison	Cohen's d				Cliff's Delta			
	D Estimation	95 CI		Effect Size	D Estimation	95 CI		Effect Size
		Lower	Upper			Lower	Upper	
Sumatera - Java	0.6993	-3.7329	5.1315	medium	0.5000	-0.8416	0.9811	large
Sumatera - Bali_Nusa Tenggara	-2.3484	-7.9407	3.2440	large	-1.0000	-1.0000	0.8283	large
Sumatera - Kalimantan	2.9904	-3.2711	9.2520	large	1.0000	-0.8283	1.0000	large
Sumatera - Sulawesi	5.6664	-3.9676	15.3005	large	1.0000	-0.8283	1.0000	large
Sumatera - Maluku	-0.0851	-4.3897	4.2195	negligible	0.0000	-0.9740	0.9740	negligible
Sumatera - Papua	-1.2963	-6.0294	3.4367	large	-0.5000	-0.9811	0.8416	large
Java - Bali_Nusa Tenggara	-4.0550	-11.5759	3.4659	large	-1.0000	-1.0000	0.8283	large
Java - Kalimantan	2.7886	-3.2536	8.8308	large	1.0000	-0.8283	1.0000	large
Java - Sulawesi	6.1032	-4.1296	16.3361	large	1.0000	-0.8283	1.0000	large
Java - Maluku	-0.5407	-4.9213	3.8398	medium	-0.2500	-0.9760	0.9347	small
Java - Papua	-2.0924	-7.4445	3.2596	large	-1.0000	-1.0000	0.8283	large
Bali_Nusa Tenggara - Kalimantan	9.8858	-5.7561	25.5277	large	1.0000	-0.8283	1.0000	large
Bali_Nusa Tenggara - Sulawesi	13.6667	-7.5639	34.8972	large	1.0000	-0.8283	1.0000	large
Bali_Nusa Tenggara - Maluku	1.2864	-3.4403	6.0132	large	1.0000	-0.8283	1.0000	large
Bali_Nusa Tenggara - Papua	0.4725	-3.8898	4.8348	small	0.0000	-0.9740	0.9740	negligible
Kalimantan - Sulawesi	4.6093	-3.6173	12.8360	large	1.0000	-0.8283	1.0000	large

Kalimantan - Maluku	-1.8720	-7.0316	3.2877	large	-1.0000	-1.0000	0.8283	large
Kalimantan - Papua	-4.3077	-12.1471	3.5316	large	-1.0000	-1.0000	0.8283	large
Sulawesi - Maluku	-3.5346	-10.4212	3.3519	large	-1.0000	-1.0000	0.8283	large
Sulawesi - Papua	-6.7049	-17.7750	4.3651	large	-1.0000	-1.0000	0.8283	large
Maluku - Papua	-0.8445	-5.3349	3.6458	large	-0.5000	-0.9811	0.8416	large

Table S3L. Effect size of odd ratios for preserved and instant food consumption variable

Island cluster comparison	Cohen's d				Cliff's Delta			
	D Estimation	95 CI		Effect Size	D Estimation	95 CI		Effect Size
		Lower	Upper			Lower	Upper	
Sumatera - Java	-1.6506	-6.6324	3.3311	large	-1.0000	-1.0000	0.8283	large
Sumatera - Bali_Nusa Tenggara	1.3644	-3.4127	6.1415	large	1.0000	-0.8283	1.0000	large
Sumatera - Kalimantan	0.3409	-3.9929	4.6746	small	0.0000	-0.9740	0.9740	negligible
Sumatera - Sulawesi	1.6927	-3.3216	6.7070	large	1.0000	-0.8283	1.0000	large
Sumatera - Maluku	0.8989	-3.6158	5.4136	large	0.5000	-0.8416	0.9811	large
Sumatera - Papua	-1.2802	-6.0031	3.4426	large	-0.5000	-0.9811	0.8416	large
Java - Bali_Nusa Tenggara	1.9577	-3.2751	7.1905	large	1.0000	-0.8283	1.0000	large
Java - Kalimantan	1.1576	-3.4914	5.8067	large	0.5000	-0.8416	0.9811	large
Java - Sulawesi	2.3829	-3.2432	8.0091	large	1.0000	-0.8283	1.0000	large
Java - Maluku	1.8385	-3.2932	6.9702	large	1.0000	-0.8283	1.0000	large
Java - Papua	0.9364	-3.5959	5.4687	large	0.5000	-0.8416	0.9811	large
Bali_Nusa Tenggara - Kalimantan	0.1791	-4.1322	4.4904	negligible	0.0000	-0.9740	0.9740	negligible
Bali_Nusa Tenggara - Sulawesi	1.3571	-3.4152	6.1294	large	1.0000	-0.8283	1.0000	large
Bali_Nusa Tenggara - Maluku	0.6415	-3.7705	5.0534	medium	0.0000	-0.9740	0.9740	negligible
Bali_Nusa Tenggara - Papua	-1.9793	-7.2308	3.2722	large	-1.0000	-1.0000	0.8283	large
Kalimantan - Sulawesi	0.3415	-3.9924	4.6754	small	0.0000	-0.9740	0.9740	negligible
Kalimantan - Maluku	0.1751	-4.1358	4.4860	negligible	0.0000	-0.9740	0.9740	negligible

Kalimantan - Papua	-0.7045	-5.1386	3.7296	medium	-0.5000	-0.9811	0.8416	large
Sulawesi - Maluku	-0.2279	-4.5445	4.0887	small	0.0000	-0.9740	0.9740	negligible
Sulawesi - Papua	-2.2443	-7.7368	3.2483	large	-1.0000	-1.0000	0.8283	large
Maluku - Papua	-1.4197	-6.2339	3.3946	large	-1.0000	-1.0000	0.8283	large

Table S3M. Effect size of odd ratios for smoking exposure variable

Island cluster comparison	Cohen's d				Cliff's Delta			
	D Estimation	95 CI		Effect Size	D Estimation	95 CI		Effect Size
		Lower	Upper			Lower	Upper	
Sumatera - Java	0.2979	-4.0286	4.6244	small	0.5000	-0.8416	0.9811	large
Sumatera - Bali_Nusa Tenggara	0.4267	-3.9246	4.7781	small	0.5000	-0.8416	0.9811	large
Sumatera - Kalimantan	0.1267	-4.1803	4.4336	negligible	0.5000	-0.8416	0.9811	large
Sumatera - Sulawesi	0.6716	-3.7507	5.0938	medium	0.5000	-0.8416	0.9811	large
Sumatera - Maluku	-0.4437	-4.7989	3.9116	small	0.0000	-0.9740	0.9740	negligible
Sumatera - Papua	0.1200	-4.1865	4.4265	negligible	0.0000	-0.9740	0.9740	negligible
Java - Bali_Nusa Tenggara	0.1086	-4.1973	4.4144	negligible	0.5000	-0.8416	0.9811	large
Java - Kalimantan	-0.1534	-4.4624	4.1556	negligible	-0.5000	-0.9811	0.8416	large
Java - Sulawesi	0.3236	-4.0071	4.6544	small	0.5000	-0.8416	0.9811	large
Java - Maluku	-0.6058	-5.0060	3.7945	medium	-0.5000	-0.9811	0.8416	large
Java - Papua	-0.2467	-4.5657	4.0723	small	0.0000	-0.9740	0.9740	negligible
Bali_Nusa Tenggara - Kalimantan	-0.2639	-4.5853	4.0574	small	-0.5000	-0.9811	0.8416	large
Bali_Nusa Tenggara - Sulawesi	0.2235	-4.0926	4.5395	small	0.5000	-0.8416	0.9811	large
Bali_Nusa Tenggara - Maluku	-0.6746	-5.0979	3.7487	medium	-0.5000	-0.9811	0.8416	large
Bali_Nusa Tenggara - Papua	-0.4103	-4.7580	3.9374	small	-0.5000	-0.9811	0.8416	large
Kalimantan - Sulawesi	0.4748	-3.8881	4.8376	small	0.5000	-0.8416	0.9811	large
Kalimantan - Maluku	-0.5067	-4.8778	3.8645	medium	-0.5000	-0.9811	0.8416	large

Kalimantan - Papua	-0.0426	-4.3457	4.2606	negligible	0.0000	-0.9740	0.9740	negligible
Sulawesi - Maluku	-0.7931	-5.2617	3.6755	medium	-0.5000	-0.9811	0.8416	large
Sulawesi - Papua	-0.7481	-5.1987	3.7025	medium	-0.5000	-0.9811	0.8416	large
Maluku - Papua	0.5240	-3.8519	4.8999	medium	0.0000	-0.9740	0.9740	negligible

Table S3N. Effect size of odd ratios for hypertension status variable

Island cluster comparison	Cohen's d				Cliff's Delta			
	D Estimation	95 CI		Effect Size	D Estimation	95 CI		Effect Size
		Lower	Upper			Lower	Upper	
Sumatera - Java	-0.1724	-2.4435	2.0988	negligible	-0.3333	-0.8502	0.5107	medium
Sumatera - Bali_Nusa Tenggara	-0.3938	-2.6827	1.8950	small	-0.3333	-0.8502	0.5107	medium
Sumatera - Kalimantan	-0.2648	-2.5416	2.0121	small	-0.3333	-0.8502	0.5107	medium
Sumatera - Sulawesi	-0.0656	-2.3332	2.2020	negligible	-0.1111	-0.8828	0.8227	negligible
Sumatera - Maluku	-0.2398	-2.5149	2.0353	small	-0.3333	-0.8502	0.5107	medium
Sumatera - Papua	0.2632	-2.0135	2.5400	small	0.3333	-0.6820	0.9097	medium
Java - Bali_Nusa Tenggara	-0.2242	-2.4983	2.0498	small	-0.3333	-0.8502	0.5107	medium
Java - Kalimantan	-0.0979	-2.3662	2.1704	negligible	-0.1111	-0.7930	0.6943	negligible
Java - Sulawesi	0.1168	-2.1521	2.3856	negligible	0.1111	-0.8227	0.8828	negligible
Java - Maluku	-0.0729	-2.3406	2.1948	negligible	-0.1111	-0.7930	0.6943	negligible
Java - Papua	0.4409	-1.8534	2.7353	small	0.3333	-0.6820	0.9097	medium
Bali_Nusa Tenggara Kalimantan	0.1216	-2.1475	2.3906	negligible	0.3333	-0.6820	0.9097	medium
Bali_Nusa Tenggara - Sulawesi	0.3496	-1.9346	2.6338	small	0.3333	-0.6820	0.9097	medium
Bali_Nusa Tenggara - Maluku	0.1464	-2.1236	2.4164	negligible	0.3333	-0.6820	0.9097	medium
Bali_Nusa Tenggara - Papua	0.6624	-1.6659	2.9907	medium	0.3333	-0.6820	0.9097	medium
Kalimantan - Sulawesi	0.2153	-2.0582	2.4889	small	0.1111	-0.8227	0.8828	negligible
Kalimantan - Maluku	0.0244	-2.2426	2.2915	negligible	-0.1111	-0.7930	0.6943	negligible

Kalimantan - Papua	0.5245	-1.7811	2.8301	medium	0.3333	-0.6820	0.9097	medium
Sulawesi - Maluku	-0.1892	-2.4612	2.0828	negligible	-0.3333	-0.8502	0.5107	medium
Sulawesi - Papua	0.3519	-1.9325	2.6364	small	0.3333	-0.6820	0.9097	medium
Maluku - Papua	0.4991	-1.8029	2.8011	small	0.3333	-0.6820	0.9097	medium
